# Global Registry of Acute Coronary Events Score Underestimates Post-Acute Coronary Syndrome Mortality among Cancer Patients

**DOI:** 10.3390/cancers15215222

**Published:** 2023-10-30

**Authors:** Chieh-Yang Koo, Huili Zheng, Li-Ling Tan, Ling-Li Foo, E’Ching Shih, Derek J. Hausenloy, Ross A. Soo, Alvin S. Wong, Arthur M. Richards, Chi-Hang Lee, Mark Y. Chan

**Affiliations:** 1 Department of Cardiology, National University Heart Centre Singapore, Singapore 119074, Singapore; 2 Clinical Research Unit, Khoo Teck Puat Hospital, Singapore 768828, Singapore; 3 Yong Loo Lin School of Medicine, National University of Singapore, Singapore 119077, Singapore; 4 National Registry of Diseases Office, Health Promotion Board, Singapore 168937, Singapore; 5 Cardiovascular & Metabolic Disorders Program, Duke-National University of Singapore Medical School, Singapore 169857, Singapore; 6 National Heart Research Institute Singapore, National Heart Centre, Singapore 169609, Singapore; 7 The Hatter Cardiovascular Institute, University College London, London WC1E 6HX, UK; 8 Cardiovascular Research Center, College of Medical and Health Sciences, Asia University, Taichung 41354, Taiwan; 9 Department of Haematology-Oncology, National University Cancer Institute of Singapore, Singapore 119074, Singapore; ross_soo@nuhs.edu.sg (R.A.S.);; 10 Christchurch Heart Institute, University of Otago, Dunedin 9016, New Zealand

**Keywords:** ischemic disease, coronary artery disease, cancer survivorship, risk prediction

## Abstract

**Simple Summary:**

Patients with previous cancer are more likely to suffer from a heart attack than those without cancer. Although risk stratification scores are commonly used both to guide management and estimate mortality after a heart attack, it is unclear if such scores can be applied to patients with previous cancer. As such, this study aimed to determine the performance of the well-established GRACE (Global Registry of Acute Coronary Events) score by combining large national cancer and heart attack databases. This study showed that although the GRACE score was more accurate in patients without cancer, it greatly underestimated the risk of death in patients with previous cancer. Specific risk stratification scores are required for patients with previous cancer who have a heart attack.

**Abstract:**

Background Patients with prior cancer are at increased risk of acute coronary syndrome (ACS) with poorer post-ACS outcomes. We aimed to ascertain if the Global Registry of Acute Coronary Events (GRACE) score accurately predicts mortality risk among patients with ACS and prior cancer. Methods We linked nationwide ACS and cancer registries from 2007 to 2018 in Singapore. A total of 24,529 eligible patients had in-hospital and 1-year all-cause mortality risk calculated using the GRACE score (2471 prior cancer; 22,058 no cancer). Results Patients with prior cancer had two-fold higher all-cause mortality compared to patients without cancer (in-hospital: 22.8% versus 10.3%, *p* < 0.001; 1-year: 49.0% vs. 18.7%, *p* < 0.001). Cardiovascular mortality did not differ between groups (in-hospital: 5.2% vs. 4.8%, *p* = 0.346; 1-year: 6.9% vs. 6.1%, *p* = 0.12). The area under the receiver operating characteristic curve of the GRACE score for prediction of all-cause mortality was less for prior cancer (in-hospital: 0.64 vs. 0.80, *p* < 0.001; 1-year: 0.66 vs. 0.83, *p* < 0.001). Among patients with prior cancer and a high-risk GRACE score > 140, in-hospital revascularization was not associated with lower cardiovascular mortality than without in-hospital revascularization (6.7% vs. 7.6%, *p* = 0.50). Conclusions The GRACE score performs poorly in risk stratification of patients with prior cancer and ACS.

## 1. Introduction

Due to improvements in cancer detection and treatment, more patients with cancer are living longer [1]. Although cancer-related outcomes continue to improve, cancer survivors are increasingly recognized to be at higher risk for cardiovascular disease, including acute coronary syndrome (ACS) [2,3,4]. However, due to a high burden of comorbidities and uncertain prognosis, best treatment practices for ACS in patients with cancer remain unclear. Percutaneous coronary intervention (PCI), which is routinely performed for ACS within the general population, has been associated with an increased risk of mortality and bleeding post-PCI in patients with cancer [5,6].

Although current guidelines advocate an invasive strategy in patients with cancer presenting with high-risk ACS, there is no clear recommendation on how best to risk stratify [7]. Guidelines advocate the use of risk stratification scores in guiding management of patients with ACS [8,9]. The Global Registry of Acute Coronary Events (GRACE) score is a well-established risk stratification score used to calculate mortality in patients presenting with ACS within the general population [10]. The European Society of Cardiology recommends the use of the GRACE score to estimate prognosis in patients with ACS and to identify suitable patients to undergo revascularization via PCI within 24 h as an early invasive strategy [9]. This is due to the GRACE score offering the best discriminative performance across other clinical risk scores [9,11]. Presently, expert consensus statements recommend using the same algorithms for diagnosis and monitoring of ACS in non-cancer patients to patients with cancer presenting with ACS [12]. Given the increased complications and poorer outcomes post-PCI observed in cancer patients, it is unclear if similar risk stratification with the GRACE score would be applicable to patients with cancer. Due to unaccounted risk variables, we hypothesized that the GRACE score would underestimate mortality in patients with cancer presenting with ACS compared to patients without cancer.

We linked data from nationwide registries to derive GRACE scores in patients presenting with ACS and compared scores in those with prior cancer to those without. Our objectives were, first, to determine if prior cancer was associated with a higher predicted risk of mortality via the GRACE score than those without cancer, and second, to compare the performance of the GRACE score in predicting mortality between the two groups of patients.

## 2. Materials and Methods

### 2.1. Study Design and Participants

Data from two national registries (Singapore Myocardial Infarction Registry and Cancer Registry) under the National Registry of Diseases Office of Singapore were combined in this population-based cohort study. [13]. In Singapore, notification of selected diseases, including cancer and ACS, is mandatory under the National Registry of Diseases Act. The Singapore Myocardial Infarction Registry is comprised of retrospectively collected records across all hospitals in Singapore of patients who presented with ACS beginning in 2007. Trained coordinators collect information, including demographics, clinical presentation, inpatient laboratory and echocardiographic parameters, and use of medications, prior to and at discharge [14]. As individual-level blood pressure and heart rate values were only available within the registry from 2017, data on blood pressure and heart rate were extracted from hospital-level databases from two centers and merged with the national registry for the years 2007 to 2016. The Cancer Registry is comprised of retrospectively collected records across all hospitals of all patients diagnosed with cancer since 1968. This database captures demographics and details on diagnosis and treatment within the first six months after diagnosis [15]. This study was approved by the local institutional review board (Domain Specific Review Board-C, National Healthcare Group, 2021/00141). Waiver of consent was granted for studies of public health importance using de-identified registry data.

Data linkage was performed between the Singapore Myocardial Infarction Registry and the Cancer Registry by using the unique National Registration Identity Card number for each patient. This determined if there was a prior diagnosis of cancer for patients presenting with ACS over 12 years from 1 January 2007 to 31 December 2018. The data were then subsequently matched with data from the Registry of Births and Deaths. As the reporting of deaths is mandatory by law in Singapore, this allowed us to obtain the survival status of all patients. All eligible patients presenting with ACS were classified into two groups for analysis: one with a prior diagnosis of cancer (prior cancer) and the other without (no cancer) forming the control group. Each patient had their individual GRACE score disease calculated at incident ACS. We calculated risk for each patient of in-hospital all-cause mortality through the GRACE score and 1-year all-cause mortality through the GRACE 2.0 score [16,17]. The GRACE score comprises eight variables: age, heart rate, systolic blood pressure, initial serum creatinine, Killip class, cardiac arrest, initial cardiac enzyme, and ST segment deviation [15]. The GRACE 2.0 score incorporates values from regression models based on these variables, which are then used to derive a sum estimate of the probability of adverse outcomes instead of a point system per the original GRACE score [17]. We excluded patients who developed cancer after their first ACS between 1 January 2007 and 31 December 2018 and patients with incomplete data resulting in incomplete calculation of an individual GRACE score. To maintain data in its original form, we excluded missing data from the analyses through case deletion without imputation. The outcomes of interest were in-hospital all-cause mortality and 1-year all-cause mortality post-ACS. STATA SE version 13 was used to perform all analyses.

### 2.2. Statistical Analysis

We compared demographics, comorbidities, and clinical characteristics for incident ACS in patients with prior cancer between patients without prior cancer using the chi-square test for categorical variables and the Wilcoxon rank-sum test for numeric variables. Among those with prior cancer, the predicted mortality in terms of GRACE score was compared across their cancer characteristics using the Kruskal–Wallis rank test and Wilcoxon rank-sum test, while the actual observed mortality was compared using the chi-square test. Aside from plotting the distribution of patients with prior cancer and those without cancer by their GRACE risk score, the Kaplan–Meier survival curves for the two groups of patients were also plotted. Area under the receiver operating characteristic curve (AUC) and Hosmer–Lemeshow goodness-of-fit test (GOF) were used to evaluate the diagnostic accuracy of the GRACE score for predicting mortality in the two groups of patients. *p*-Values < 0.05 were considered statistically significant.

## 3. Results

### 3.1. Baseline Demographics

**Figure 1:**
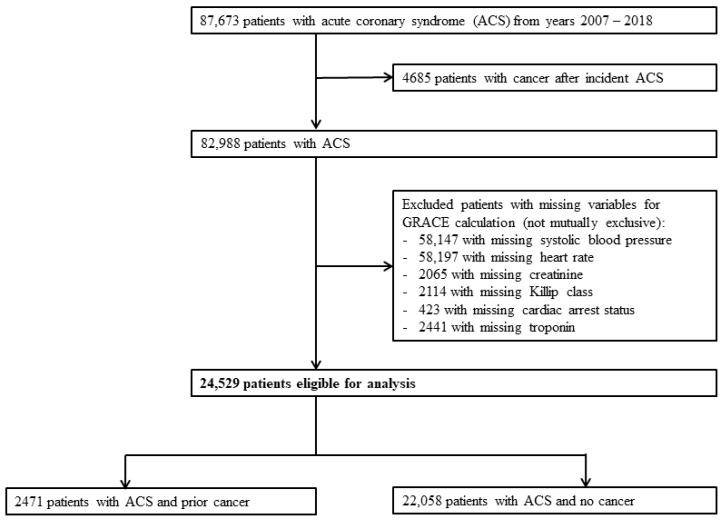
Flowchart of study patients. Study flowchart demonstrating eventual number of eligible subjects for analysis after exclusion. ACS: acute coronary syndrome.

We identified 87,673 patients with ACS between 1 January 2007 and 31 December 2018. After excluding patients who developed cancer after incident ACS, 82,988 patients were available for analysis. We then further excluded patients with incomplete data for the calculation of GRACE score. A total of 24,529 patients were available for final analysis, of which 2471 patients had prior cancer (prior-cancer group) and 22,058 patients did not have cancer (no-cancer group). (Figure 1) The median duration from cancer diagnosis to ACS was 1911 days (5 years and 85 days) among those with prior cancer.

Baseline characteristics of the two groups with incident ACS are presented in Table 1. The prior-cancer group was older, with a lower proportion of men, higher proportion of Chinese ethnicity, and a lower median body mass index. The proportion of current smokers was lower in the prior-cancer group. The prevalence of hypertension was higher in the prior-cancer group, but the median systolic and diastolic blood pressures were lower for incident ACS. The prevalence of hyperlipidemia was lower in the prior-cancer group, where the median total cholesterol and low-density lipoprotein cholesterol concentrations were also lower for incident ACS. There was no significant difference in the prevalence of diabetes mellitus or history of myocardial infarction and revascularization between the two groups.

The proportions of ST-segment elevation myocardial infarctions and cardiac arrests were lower in the prior-cancer group, and they were less likely to undergo revascularization (Table 1). Although the prior-cancer group had a lower proportion of heart failure (Killip class II, III or IV), there was no significant difference in the proportion of cardiogenic shock or median left ventricular ejection fraction. At the point of discharge after incident ACS, the prior-cancer group was less likely to be prescribed with guideline directed medical therapy.

The predicted risk from GRACE score for both in-hospital and 1-year all-cause mortality were significantly higher in the prior-cancer group compared to the no-cancer group (Table 1). Similarly, the observed in-hospital and 1-year all-cause mortality was also significantly higher in the prior-cancer group. However, there was no significant difference in in-hospital and 1-year cardiovascular mortality.

### 3.2. Predicted and Observed All-Cause Mortality by Cancer Status

**Figure 2:**
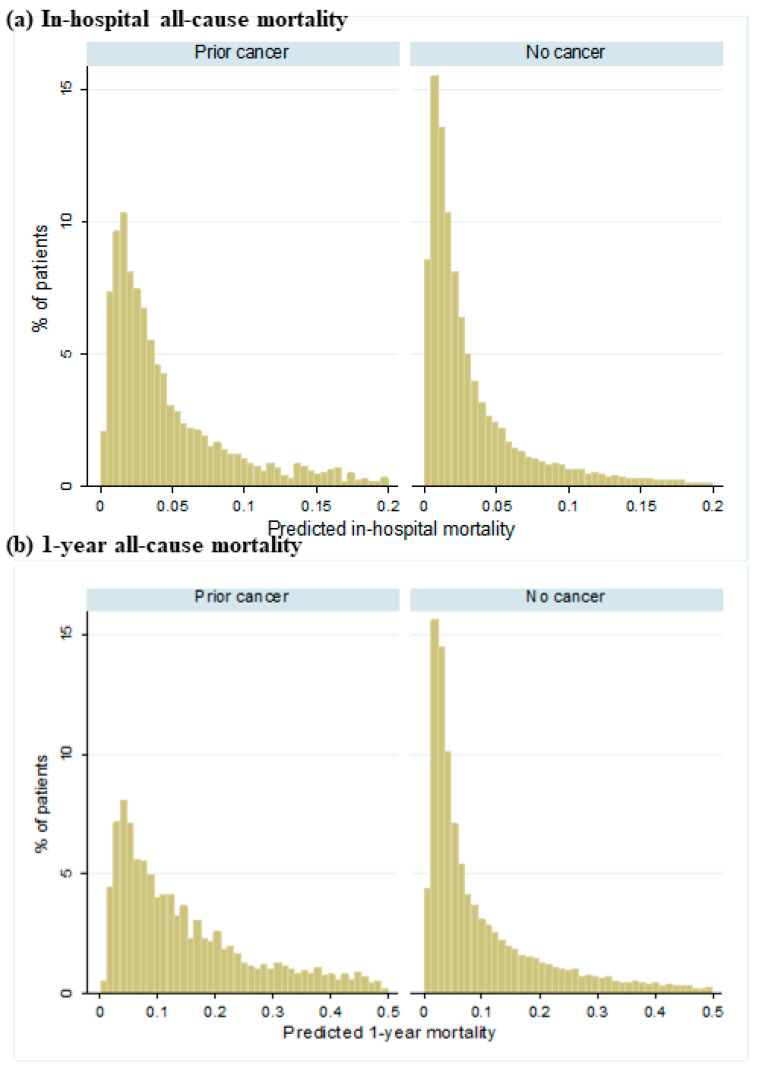
Distribution of predicted all-cause mortality by cancer status. (**a**) demonstrates the predicted in-hospital all-cause mortality between patients with prior cancer and no cancer. (**b**) demonstrates the predicted 1-year all-cause mortality between patients with prior cancer and no cancer.

**Figure 3:**
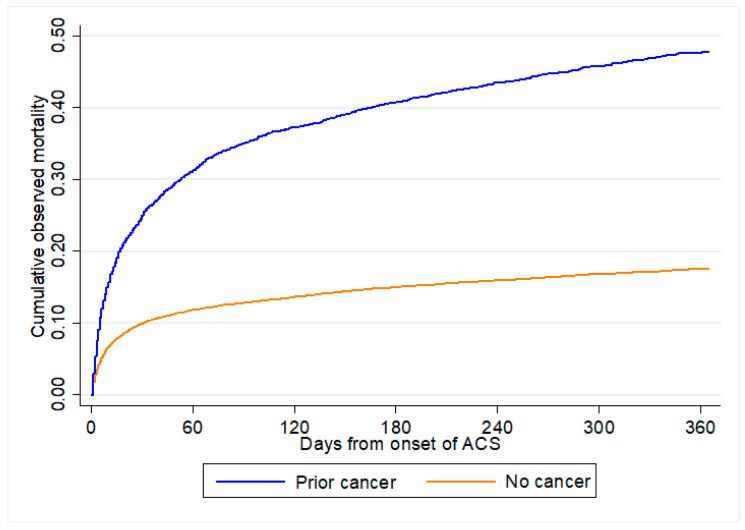
Observed all-cause mortality by cancer status. All-cause mortality comparing between patients with prior cancer and no cancer from onset of ACS. ACS: acute coronary syndrome.

Figure 2 shows the distribution of predicted all-cause mortality by cancer status. A greater proportion of patients with prior cancer were predicted to have higher risk for both in-hospital mortality and 1-year all-cause mortality than those without cancer. Figure 3 shows the cumulative observed all-cause mortality by cancer status. Observed all-cause mortality was higher in those with prior cancer than no cancer regardless of the post-ACS period.

### 3.3. Performance of GRACE Score by Cancer Status

**Figure 4:**
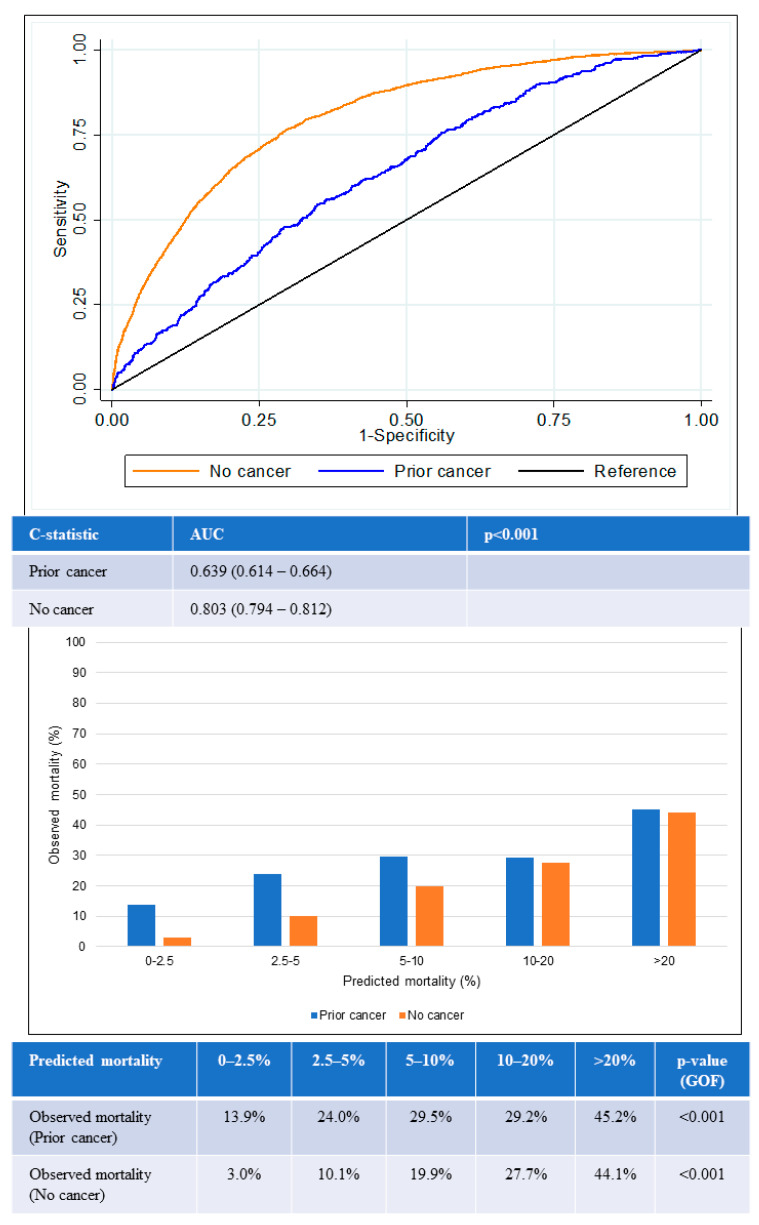
Performance of GRACE score for in-hospital mortality according to cancer status. Receiver operating curves (upper) showing AUC for the GRACE score according to cancer status. Chart (lower) comparing predicted mortality against observed mortality according to cancer status. AUC: area under the receiver operating curve; GOF: goodness of fit.

The AUC for in-hospital all-cause mortality among patients with prior cancer was 0.64 (95% confidence interval [CI] 0.61–0.66), which was significantly lower than the AUC among those with no cancer (0.80, 95% CI 0.79–0.81) (*p* < 0.001) (Figure 4). The lower AUC for the prior-cancer group indicated that the GRACE score was poorer in differentiating the mortality status of patients with prior cancer. The GOF test for in-hospital all-cause mortality was statistically significant in both groups of patients, suggesting that predicted mortality risk from GRACE score was not close to the observed mortality regardless if the patients had prior cancer or no cancer.

**Figure 5:**
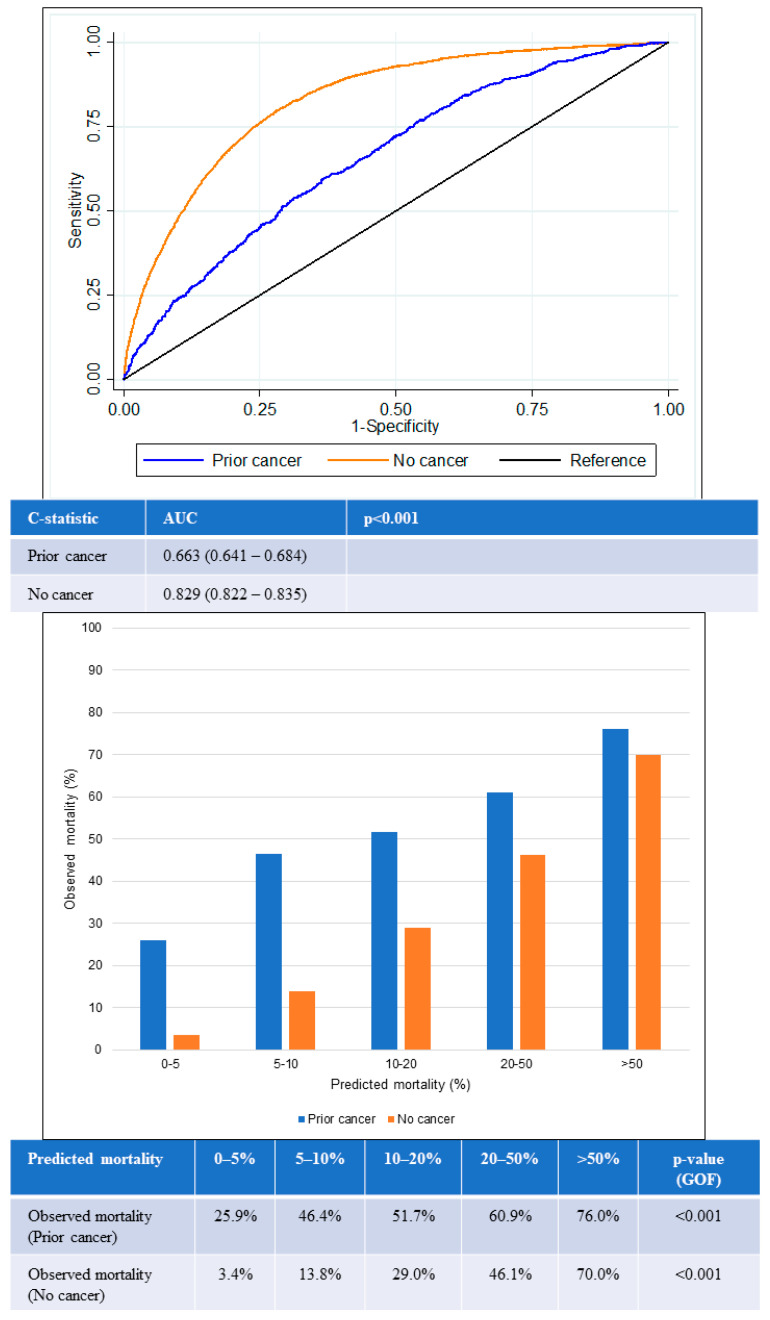
Performance of GRACE score for 1-year mortality according to cancer status. Receiver operating curves (upper) showing AUC for the GRACE score according to cancer status. Chart (lower) comparing predicted mortality against observed mortality according to cancer status. AUC: area under the receiver operating curve; GOF: goodness of fit.

Likewise, similar trends were observed for 1-year all-cause mortality (Figure 5). The AUC for the prior-cancer group was 0.66 (95% CI 0.64–0.68), while the AUC for the no-cancer group was 0.83 (95% CI 0.82–0.84). This was again significantly lower in the prior-cancer group (*p* < 0.001), indicating poorer discrimination by the GRACE score among patients in this group. The GOF test for 1-year all-cause mortality was again statistically significant in both groups of patients.

The GRACE score showed suboptimal calibration among both prior cancer and no-cancer groups. Underestimation of mortality in the prior-cancer group was worse than for the no-cancer group across all quintiles of risk. The greatest underestimation of mortality was observed in the lowest two quintiles of risk in the prior-cancer group in which observed mortality exceeded predicted mortality by two- to six-fold in the lower two quintiles of risk.

### 3.4. Mortality by Revascularization Status for Low and High GRACE Score

In-hospital revascularization was associated with lower in-hospital and 1-year all-cause mortality regardless of cancer status and GRACE score (Table 2). In-hospital revascularization was not associated with lower cardiovascular mortality in the prior-cancer group but was instead associated with lower cardiovascular mortality in the no-cancer group.

When classified according to the GRACE score, in-hospital revascularization in the prior-cancer group with a high-risk GRACE score (>140) was not associated with lower cardiovascular mortality but was associated with lower 1-year cardiovascular mortality in the no-cancer group with a high-risk GRACE score (Table 2).

### 3.5. Predicted and Observed Mortality by Cancer Characteristics among Those with Prior Cancer

Table 3 shows the predicted and observed in-hospital all-cause mortality by cancer characteristics among those with prior cancer. While there was no significant difference in predicted in-hospital all-cause mortality across cancer stages, observed mortality increased significantly with cancer stage. Predicted in-hospital all-cause mortality was lowest in those most recently diagnosed with cancer, but observed in-hospital all-cause mortality was highest in this group. Head and neck cancer patients were predicted to have the lowest in-hospital all-cause mortality, while skin cancer patients were predicted to have the highest in-hospital all-cause mortality. Observed in-hospital all-cause mortality was lowest in thyroid cancer patients and highest in lung cancer patients. Predicted in-hospital all-cause mortality was lower for most patients who received treatment for cancer. However, aside from lower observed in-hospital all-cause mortality in patients who underwent surgery than those who did not, there was no other significant difference in observed in-hospital all-cause mortality by cancer treatment.

Table 4 shows the predicted and observed 1-year all-cause mortality by cancer characteristics among those with prior cancer. Predicted 1-year all-cause mortality was highest in early stage cancer, but observed 1-year all-cause mortality increased with cancer stage. Predicted 1-year all-cause mortality was lowest in those most recently diagnosed with cancer, but observed 1-year all-cause mortality was again highest in this group. When classified according to cancer subtype or cancer treatment, the 1-year all-cause mortality trends were similar to that of in-hospital all-cause mortality.

Specific to cardiovascular mortality, both in-hospital and 1-year cardiovascular mortality was significantly lower in patients recently diagnosed with cancer (within the first quartile of 425 days) compared to patients with a longer interval from cancer diagnosis (across the other three quartiles) (Table 5). When classified according to cancer stage or cancer therapy received, there was no significant difference in cardiovascular mortality.

## 4. Discussion

This study compared the performance of the GRACE score in prediction of all-cause mortality after ACS among patients with and without prior cancer. The AUC of the GRACE score was substantially lower in the prior-cancer group compared with the no-cancer group for both in-hospital all-cause mortality (0.64 vs. 0.80, *p* < 0.001) and 1-year all-cause mortality (0.66 vs. 0.83, *p* < 0.001). The GRACE score performed worse among the lowest two quintiles of risk, in which observed mortality was two- to six-fold higher than predicted mortality. In patients with high GRACE scores, in-hospital revascularization was not associated with lower cardiovascular mortality in the prior-cancer group but was associated with lower cardiovascular mortality in the no-cancer group. Our findings highlight that patients with cancer who develop ACS have a very high risk of mortality that is not accurately predicted by a conventional ACS risk score.

Several reasons can account for the poorer performance of the GRACE score in patients with prior cancer. Firstly, the GRACE score does not account for the cardiotoxic effects of cancer therapies. Cancer therapies are independently associated with increased risk of cardiovascular toxicities [18,19]. These include common associations such as hypertension from tyrosine kinase inhibitors, heart failure from anthracyclines and trastuzumab, vasospasm from 5-fluorouracil and its derivatives, ischemia from vascular endothelial growth factor inhibition, and even myocarditis and accelerated atherosclerosis from newer agents such as immune-checkpoint inhibitors [20,21,22]. Radiotherapy can also lead to atherosclerosis as well as valvular, myocardial, and pericardial dysfunction [23].

Secondly, the GRACE score does not include cancer characteristics when predicting all-cause mortality. This was most evident through our findings where patients with advanced stage cancer were conversely of a lower predicted risk of mortality than those with early stage cancer when the GRACE score was applied. This was despite the markedly elevated observed mortality in those with advanced stage cancer. Other cancer characteristics such as cancer subtypes may be of similar relevance although more research will be required. For example, we identified patients with thyroid cancer to have the lowest in-patient all-cause mortality and those with head and neck cancer to have the lowest 1-year all-cause mortality. Patients with lung cancer had the highest in-patient and 1-year all-cause mortality. This is consistent with other studies also demonstrating highest mortality in patients with lung cancer post-myocardial infarction or post-PCI [24,25]. We also identified that patients with a recent diagnosis of cancer, within 425 days (first quartile within the study population), had the highest all-cause mortality but lowest cardiovascular mortality when compared to those with a cancer diagnosis beyond this period. This may suggest that in patients with an early diagnosis of cancer within 1 year, especially lung cancer, the benefit of an early invasive revascularization strategy may be less pronounced. The GRACE score could therefore be further optimized by incorporating cancer characteristics, such as cancer type, staging, and duration, from cancer diagnosis to better guide revascularization strategies in patients with cancer.

Thirdly, there are significant treatment biases which exist when treating prior cancer patients with ACS. Similar to other studies, we found that patients with prior cancer were less likely to undergo revascularization or receive guideline-directed medical therapy compared to patients without a history of cancer [25,26,27]. These treatment biases would inadvertently lead to the increased mortality in patients with prior cancer unaccounted for by the GRACE score. Increased education and awareness in both patients and medical practitioners are required to address this gap in guideline directed care within this challenging and often neglected patient population.

Therefore, risk stratification and management of the patient with cancer with ACS currently lacks a strong scientific evidence base. Guidelines recommend pursuing an early invasive strategy for revascularization guided by use of the GRACE score [9]. However, our findings demonstrate that this grossly underestimated mortality risk in patients with cancer. Although revascularization was associated with lower all-cause mortality in prior cancer patients, this occurred regardless of GRACE score. This observed lower all-cause mortality is likely due to selection bias similar to other studies, where those with a better prognosis were more likely to undergo revascularization [25]. However, among high-risk patients with prior cancer identified via the GRACE score, in-hospital revascularization did not reduce cardiovascular mortality compared to those who did not undergo revascularization. Hence, the benefit of pursuing an aggressive revascularization strategy in patients with prior cancer guided by the GRACE score may not be unequivocally beneficial. The expected benefit of revascularization in cancer patients could be reduced by an overall poor prognosis from cancer or possible increased thrombotic or bleeding complications after revascularization in prior cancer patients [5,6,28,29]. We suggest adopting a more individualized approach to revascularization in prior cancer patients, similar to how cancer patients are risk assessed prior to commencement of cancer treatment [30]. In the absence of a well validated risk stratification tool specific to prior cancer patients, we propose a multi-disciplinary approach involving the cancer physician, cardio-oncologist, and interventional cardiologist to guide revascularization decisions.

To the best our knowledge, this is the first study demonstrating the performance of the GRACE score in patients with both ACS and cancer. However, there are several limitations to our study. First, data were not available on the etiology of ACS as we were not able to retrieve details on whether the ACS was secondary to atherosclerosis or complications from cancer therapy, such as vasospasm. Second, data on PCI characteristics, such as the use of balloon angioplasty or choice of stents deployed, the use of intravascular imaging to optimize PCI outcomes in cancer patients which have been recommended by guidelines, and the duration of dual anti-platelet therapy, were all not available [31]. Third, the GRACE score was developed in a predominantly European population and not a multiethnic Asian population. However, our results remained similar despite recalibration of the GRACE score for use within the local population [32].

## 5. Conclusions

In conclusion, the GRACE score performed much worse among patients with ACS and prior cancer than patients without cancer. Only a minority of patients with cancer underwent in-hospital revascularization. Among patients identified as high-risk through the GRACE score, in-hospital revascularization was not associated with lower cardiovascular mortality in patients with prior cancer but was associated with lower 1-year cardiovascular mortality in patients with no cancer. Further research is needed to identify other risk variables unique to this special population to better risk stratify and guide treatment strategies for patients with prior cancer and ACS.

## Figures and Tables

**Table 1:** Demographic and clinical characteristics of patients with incident ACS.

	Prior Cancer (*n* = 2471)	No Cancer (*n* = 22,058)	*p*-Value
Age, median (IQR), years	76 (67–84)	64 (55–76)	<0.001
Male sex, *n* (%)	1405 (56.9)	15,999 (72.5)	<0.001
Ethnicity, *n* (%)			<0.001
Chinese	1996 (80.8)	14,188 (64.3)	
Malay	275 (11.1)	4580 (20.8)	
Indian	182 (7.4)	2981 (13.5)	
Others	18 (0.7)	309 (1.4)	
Body mass index, median (IQR), kg/m^2^	22.7 (19.8–25.5)	24.4 (21.9–27.4)	<0.001
Cardiovascular risk factors			
Current smoker, *n* (%)	273 (11.1)	6978 (31.6)	<0.001
Hypertension, *n* (%)	1883 (76.2)	14,658 (66.5)	<0.001
Systolic blood pressure, median (IQR), mmHg	131 (110–152)	134 (115–155)	<0.001
Diastolic blood pressure, median (IQR), mmHg	69 (59–80)	75 (63–88)	<0.001
Hyperlipidaemia, *n* (%)	1591 (64.4)	14,978 (67.9)	<0.001
Total cholesterol, median (IQR), mmol/L	4.16 (3.31–5.05)	4.85 (4.00–5.74)	<0.001
HDL cholesterol, median (IQR), mmol/L	1.10 (0.89–1.32)	1.06 (0.90–1.26)	0.12
LDL cholesterol, median (IQR), mmol/L	2.45 (1.70–3.26)	3.08 (2.31–3.90)	<0.001
Diabetes mellitus, *n* (%)	1097 (44.4)	9854 (44.7)	0.79
HbA1c, median (IQR), %	6.1 (5.6–7.2)	6.1 (5.6–7.6)	0.002
History of ACS or revascularization, *n* (%)	344 (13.9)	2785 (12.6)	0.067
ACS characteristics			
STEMI, *n* (%)	381 (18.7)	7776 (37.9)	<0.001
Cardiac arrest, *n* (%)	42 (1.7)	526 (2.4)	0.032
Killip class on arrival, *n* (%)			0.003
I	2106 (85.2)	18,185 (82.4)	
II	165 (6.7)	1595 (7.2)	
III	140 (5.7)	1581 (7.2)	
IV	60 (2.4)	697 (3.2)	
Heart rate, median (IQR), beats per minute	89 (74–105)	82 (69–99)	<0.001
Creatinine, median (IQR), µmol/L	101 (73–155)	91 (75–124)	<0.001
Underwent revascularization, *n* (%)	606 (24.5)	12,714 (57.6)	<0.001
LVEF, median (IQR), %	46 (35–59)	48 (35–60)	0.18
Medications at discharge			
Aspirin, *n* (%)	1297 (68.3)	17,293 (87.6)	<0.001
Beta-blocker, *n* (%)	1304 (68.6)	16,082 (81.4)	<0.001
ACE-I/ARB, *n* (%)	795 (41.8)	12,154 (61.5)	<0.001
Lipid lowering therapy, *n* (%)	1467 (77.2)	18,350 (92.9)	<0.001
Mortality			
Predicted risk for in-hospital mortality, median (IQR), %	3.3 (1.7–7.0)	2.1 (1.0–4.9)	<0.001
Observed in-hospital mortality, *n* (%)	564 (22.8)	2261 (10.3)	<0.001
Observed in-hospital cardiovascular mortality, *n* (%)	129 (5.2)	1057 (4.8)	0.35
Predicted risk for 1-year mortality, median (IQR), %	12.1 (5.7–23.8)	5.9 (2.9–15.3)	<0.001
Observed 1-year mortality, *n* (%)	1211 (49.0)	4129 (18.7)	<0.001
Observed 1-year cardiovascular mortality, *n* (%)	170 (6.9)	1342 (6.1)	0.12

Abbreviations: ACE-I: angiotensin-converting enzyme inhibitor; ACS: acute coronary syndrome; ARB: angiotensin II receptor blocker; HDL: high density lipoprotein cholesterol; IQR: interquartile range; LDL: low-density lipoprotein cholesterol; LVEF: left ventricular ejection fraction; STEMI: ST-segment elevation myocardial infarction. Unknown values were excluded.

**Table 2:** Mortality by in-hospital revascularization status in patients with prior cancer. (A) Among Those with Prior Cancer; (B) Among Those with No Cancer.

**(A)**			
**Among Those with Prior Cancer**			
	**No Revascularization ** **(*n* = 1865)**	**With Revascularization** **(*n* = 606)**	***p*** **-Value**
In-hospital all-cause mortality, *n* (%)	516 (27.7)	48 (7.9)	<0.001
In-hospital cardiovascular mortality, *n* (%)	101 (5.4)	28 (4.6)	0.45
1-year all-cause mortality, *n* (%)	1107 (59.4)	104 (17.2)	<0.001
1-year cardiovascular mortality, *n* (%)	136 (7.3)	34 (5.6)	0.16
Among those with prior cancer and GRACE score ≤ 140			
	No revascularization (*n* = 815)	With revascularization (*n* = 320)	*p*-value
In-hospital all-cause mortality, *n* (%)	178 (21.8)	4 (1.3)	<0.001
In-hospital cardiovascular mortality, *n* (%)	31 (3.8)	2 (0.6)	0.003
1-year all-cause mortality, *n* (%)	35 (33.0)	4 (4.2)	<0.001
1-year cardiovascular mortality, *n* (%)	3 (2.8)	0 (0.0)	0.25
Among those with prior cancer and GRACE score > 140			
	No revascularization (*n* = 1050)	With revascularization (*n* = 286)	*p*-value
In-hospital all-cause mortality, *n* (%)	338 (32.2)	44 (15.4)	<0.001
In-hospital cardiovascular mortality, *n* (%)	70 (6.7)	26 (9.1)	0.16
1-year all-cause mortality, *n* (%)	1072 (60.9)	100 (19.6)	<0.001
1-year cardiovascular mortality, *n* (%)	133 (7.6)	34 (6.7)	0.50
**(B)**			
**Among Those with No Cancer**			
	**No Revascularization ** **(*n* = 9344)**	**With Revascularization** **(*n* = 12714)**	***p*** **Value**
In-hospital all-cause mortality, *n* (%)	1672 (17.9)	589 (4.6)	<0.001
In-hospital cardiovascular mortality, *n* (%)	620 (6.6)	437 (3.4)	<0.001
1-year all-cause mortality, *n* (%)	3184 (34.1)	945 (7.4)	<0.001
1-year cardiovascular mortality, *n* (%)	835 (8.9)	507 (4.0)	<0.001
Among those with no cancer and GRACE score ≤ 140			
	No revascularization (*n* = 4436)	With revascularization (*n* = 9213)	*p*-value
In-hospital all-cause mortality, *n* (%)	381 (8.6)	74 (0.8)	<0.001
In-hospital cardiovascular mortality, *n* (%)	107 (2.4)	37 (0.4)	<0.001
1-year all-cause mortality, *n* (%)	87 (7.1)	44 (0.9)	<0.001
1-year cardiovascular mortality, *n* (%)	15 (1.2)	13 (0.3)	<0.001
Among those with no cancer and GRACE score > 140			
	No revascularization (*n* = 4908)	With revascularization (*n* = 3501)	*p*-value
In-hospital all-cause mortality, *n* (%)	1291 (26.3)	515 (14.7)	<0.001
In-hospital cardiovascular mortality, *n* (%)	513 (10.5)	400 (11.4)	0.16
1-year all-cause mortality, *n* (%)	3097 (38.2)	901 (11.3)	<0.001
1-year cardiovascular mortality, *n* (%)	820 (10.1)	494 (6.2)	<0.001

Abbreviations: GRACE: Global Registry of Acute Coronary Events.

**Table 3:** In-hospital all-cause mortality by cancer characteristics in patients with prior cancer.

	Predicted Risk in %, Median (IQR)	*p* Value	Observed Mortality, *n* (%)	*p*-Value
* Cancer AJCC staging		0.568		<0.001
I	3.5 (1.6–7.6)		203 (54.1)	
II	3.1 (1.7–7.1)		207 (57.2)	
III	3.0 (1.7–5.7)		205 (65.1)	
IV	2.9 (1.6–6.6)		298 (83.9)	
^†^ Duration from cancer diagnosis		<0.001		<0.001
1st quartile	2.8 (1.5–5.7)		469 (75.9)	
2nd quartile	3.4 (1.6–7.4)		396 (64.1)	
3rd quartile	3.6 (1.8–8.0)		364 (58.9)	
4th quartile	3.6 (1.8–7.2)		324 (52.5)	
Cancer subtype		<0.001		<0.001
Head and neck	2.5 (1.2–4.4)		83 (51.9)	
Upper gastrointestinal	3.8 (2.1–6.9)		75 (69.4)	
Hepatobiliary and pancreas	3.0 (1.7–6.3)		123 (82.6)	
Colorectal and anal	3.5 (1.7–8.5)		262 (59.0)	
Lung and pleura	3.1 (1.7–6.0)		140 (88.1)	
Thyroid	3.0 (1.3–7.3)		20 (50.0)	
Breast	3.6 (1.8–7.1)		156 (56.9)	
Gynecological	2.9 (1.4–6.1)		113 (52.6)	
Urological (Kidney and bladder)	3.8 (1.7–9.8)		102 (60.4)	
Prostate	3.7 (1.8–7.6)		139 (60.4)	
Hematological	3.3 (1.7–6.1)		163 (65.7)	
Skin (Melanoma and non-melanoma)	4.5 (2.3–7.9)		129 (67.2)	
^‡^ Cancer treatment				
Surgery	3.1 (1.6–6.7)	0.075	510 (55.0)	<0.001
No surgery	3.4 (1.7–7.0)		723 (76.0)	
Radiotherapy	2.6 (1.4–5.1)	<0.001	217 (62.5)	0.18
No radiotherapy	3.4 (1.7–7.3)		1016 (66.3)	
Chemotherapy	2.6 (1.4–5.0)	<0.001	342 (66.7)	0.56
No chemotherapy	3.5 (1.8–7.7)		891 (65.2)	
Hormone therapy	3.8 (1.9–8.1)	0.006	175 (65.5)	0.98
No hormone therapy	3.1 (1.6–6.7)		1058 (65.6)	
Biological therapy	2.8 (1.6–5.2)	0.159	58 (61.1)	0.37
No biological therapy	3.2 (1.7–7.0)		1175 (65.9)	

Abbreviations: AJCC: American Joint Committee on Cancer; IQR: interquartile range. * Data on cancer staging are only available for cancer cases diagnosed from 2003 onwards. ^†^ Quartiles: 0–425 days, 426–1911 days, 1912–4820 days, >4820 days. ^‡^ Limited to treatment received within the first 6 months from cancer diagnosis and data on treatment are only available for cancer cases diagnosed from 2003 onwards.

**Table 4:** One-year all-cause mortality by cancer characteristics in patients with prior cancer.

	Predicted Risk in %, Median (IQR)	*p* Value	Observed Mortality, *n* (%)	*p*-Value
* Cancer AJCC staging		0.021		<0.001
I	14.3 (5.8–26.3)		143 (38.1)	
II	10.9 (5.5–23.0)		145 (40.1)	
III	11.2 (5.6–21.1)		171 (54.3)	
IV	9.9 (5.1–19.5)		266 (74.9)	
^†^ Duration from cancer diagnosis		<0.001		<0.001
1st quartile	9.7 (5.3–19.5)		411 (66.5)	
2nd quartile	12.0 (5.6–23.5)		311 (50.3)	
3rd quartile	13.9 (6.1–27.4)		251 (40.6)	
4th quartile	13.0 (6.7–26.4)		238 (38.6)	
Cancer subtype		<0.001		<0.001
Head and neck	7.0 (3.8–15.0)		56 (35.0)	
Upper gastrointestinal	12.6 (6.7–23.0)		68 (63.0)	
Hepatobiliary and pancreas	10.4 (5.6–21.3)		109 (73.2)	
Colorectal and anal	13.1 (6.3–29.2)		197 (44.4)	
Lung and pleura	10.6 (5.9–19.4)		123 (77.4)	
Thyroid	11.8 (4.1–31.1)		13 (38.2)	
Breast	12.4 (6.3–23.8)		109 (39.8)	
Gynaecological	10.9 (5.1–22.9)		92 (42.8)	
Urological (Kidney and bladder)	14.4 (6.4–31.7)		81 (47.9)	
Prostate	13.9 (7.3–28.3)		100 (43.5)	
Haematological	11.6 (4.8- 20.2)		132 (53.2)	
Skin (Melanoma and non-melanoma)	18.0 (9.5–31.4)		91 (47.4)	
^‡^ Cancer treatment				
Surgery	11.1 (5.3–23.3)	0.126	387 (41.7)	<0.001
No surgery	12.4 (5.7–23.3)		603 (63.4)	
Radiotherapy	8.6 (4.4–18.0)	<0.001	175 (50.4)	0.35
No radiotherapy	12.6 (5.9–24.7)		815 (53.2)	
Chemotherapy	8.0 (4.4–16.9)	<0.001	281 (54.8)	0.27
No chemotherapy	13.2 (6.5–26.2)		709 (51.9)	
Hormone therapy	13.3 (6.6–28.3)	0.016	134 (50.2)	0.38
No hormone therapy	11.3 (5.4–23.0)		856 (53.1)	
Biological therapy	7.4 (4.7–16.3)	0.004	46 (48.4)	0.39
No biological therapy	11.9 (5.6–23.7)		944 (52.9)	

Abbreviations: AJCC: American Joint Committee on Cancer; IQR: interquartile range. * Data on cancer staging are only available for cancer cases diagnosed from 2003 onwards. ^†^ Quartiles: 0–425 days, 426–1911 days, 1912–4820 days, >4820 days. ^‡^ Limited to treatment received within the first 6 months from cancer diagnosis and data on treatment are only available for cancer cases diagnosed from 2003 onwards.

**Table 5:** Cardiovascular mortality by cancer characteristics in patients with prior cancer.

	In-Hospital Cardiovascular Mortality, *n* (%)	*p* Value	1-Year Cardiovascular Mortality, *n* (%)	*p*-Value
* Cancer AJCC staging		0.175		0.45
I	26 (6.9)		29 (7.7)	
II	16 (4.4)		22 (6.1)	
III	11 (3.5)		17 (5.4)	
IV	16 (4.5)		18 (5.1)	
^†^ Duration from cancer diagnosis		0.050		0.025
1st quartile	19 (3.1)		26 (4.2)	
2nd quartile	38 (6.2)		49 (7.9)	
3rd quartile	37 (6.0)		49 (7.9)	
4th quartile	35 (5.7)		46 (7.5)	
^‡^ Cancer treatment				
Radiotherapy	17 (4.9)	0.806	22 (6.3)	0.73
No radiotherapy	80 (5.2)		105 (6.9)	
Chemotherapy	23 (4.5)	0.415	33 (6.4)	0.73
No chemotherapy	74 (5.4)		94 (6.9)	
Hormone therapy	12 (4.5)	0.594	12 (4.5)	0.11
No hormone therapy	85 (5.3)		115 (7.1)	
Biological therapy	3 (3.2)	0.365	3 (3.2)	0.15
No biological therapy	94 (5.3)		124 (7.0)	
At least one of the four therapies	40 (4.6)	0.320	54 (6.2)	0.40
None of the four therapies	57 (5.6)		73 (7.2)	

Abbreviations: AJCC: American Joint Committee on Cancer; IQR: interquartile range. * Data on cancer staging are only available for cancer cases diagnosed from 2003 onwards. ^†^ Quartiles: 0–425 days, 426–1911 days, 1912–4820 days, >4820 days. ^‡^ Limited to treatment received within the first 6 months from cancer diagnosis and data on treatment are only available for cancer cases diagnosed from 2003 onwards.

## Data Availability

The data presented in this study are available on request from the corresponding author. The patient-identifiable data are not publicly available as they are under the jurisdiction of the Ministry of Health Singapore.
